# Imaging of skull vault tumors in adults

**DOI:** 10.1186/s13244-019-0820-9

**Published:** 2020-02-13

**Authors:** Albert Pons Escoda, Pablo Naval Baudin, Paloma Mora, Mònica Cos, Javier Hernandez Gañan, José A. Narváez, Carles Aguilera, Carles Majós

**Affiliations:** 1 Department of Neuroradiology, Hospital Universitari de Bellvitge, C. Feixa Llarga SN, 08907 L’Hospitalet de Llobregat, Spain; 2 Department of Musculoskeletal Radiology, Hospital Universitari de Bellvitge, C. Feixa Llarga SN, 08907 L’Hospitalet de Llobregat, Spain

**Keywords:** Skull neoplasms, Magnetic resonance imaging, Computed tomography, Perfusion imaging, Magnetic resonance spectroscopy

## Abstract

The skull vault, formed by the flat bones of the skull, has a limited spectrum of disease that lies between the fields of neuro- and musculoskeletal radiology. Its unique abnormalities, as well as other ubiquitous ones, present particular features in this location. Moreover, some benign entities in this region may mimic malignancy if analyzed using classical bone-tumor criteria, and proper patient management requires being familiar with these presentations. This article is structured as a practical review offering a systematic diagnostic approach to focal calvarial lesions, broadly organized into four categories: (1) pseudolesions: arachnoid granulations, meningo-/encephaloceles, vascular canals, frontal hyperostosis, parietal thinning, parietal foramina, and sinus pericrani; (2) lytic: fibrous dysplasia, epidermal inclusion and dermoid cysts, eosinophilic granuloma, hemangioma, aneurysmal bone cyst, giant cell tumor, metastasis, and myeloma; (3) sclerotic: osteomas, osteosarcoma, and metastasis; (4) transdiploic: meningioma, hemangiopericytoma, lymphoma, and metastasis, along with other less common entities. Tips on the potential usefulness of functional imaging techniques such as MR dynamic susceptibility (T2*) perfusion, MR spectroscopy, diffusion-weighted imaging, and PET imaging are provided.

## Key points

- The skull vault has its own limited spectrum of disease.
- Pseudolesions should be known and easily recognized.
- Some benign entities may mimic malignancy if analyzed using classical signs.
- Recognition of key findings may assist in the differential diagnosis.
- MR spectroscopy, MR diffusion, and MR perfusion curve analysis may be useful for specific scenarios.

## Background

Most reviews of bone lesions in the head-and-neck region include skull base, face, and spine [1–3], whilst to our knowledge, there are fewer practical pictorial reviews specifically focused on skull *vault* lesions. However, the skull vault deserves attention, since it has its own limited spectrum of disease with some unique entities and other ubiquitous ones, which may present specific features in this location.

Anatomically, the skull vault or calvarium is formed by the convex part of the neurocranium composed by flat bones: both frontal bones, both parietal bones, the squamous part of both temporal bones, and the interparietal part of the occipital bone. These are embryologically formed by intramembranous ossification, in contrast to the skull base (chondrocranium), which is formed by endochondral ossification [4].

Skull vault lesions are often found incidentally on cranial imaging. Therefore, lack of clinical information or nonspecific presentation may further complicate characterization. In this context, confounding pseudolesions are frequent in this location (e.g., arachnoid granulations, vascular canals). Moreover, specific benign lesions (e.g., meningioma, hemangioma) may have an apparently aggressive bone-involvement, if analyzed using classical imaging signs used for other extracranial bones.

The purpose of this review is to serve as a practical pictorial reference of the range of lesions the radiologist might encounter. Thus, abnormalities have been broadly classified according to their major components: lytic, sclerotic, or transdiploic (with soft-tissue components overcoming calvarial tables) (Table 1). Additional signs such as periosteal reaction, cortical bone expansion, transition zone, bone sequestrum, bone permeation, soft-tissue features, and enhancement, as well as relevant clinical and demographical information, have been considered. Finally, scenarios in which functional imaging techniques such as MR spectroscopy, MR diffusion, MR perfusion curve analysis, and PET imaging can offer relevant clues have been included.

**Table 1:** Classification by pattern of bone involvement

Pseudolesions	Lytic	Sclerotic/mixed	Transdiploic
Arachnoid granulation Meningo-/encephalocele Dilated vascular canals Internal hyperostosis Biparietal thinning Enlarged parietal foramina Sinus pericrani	Fibrous dysplasia Epidermal inclusion cyst Eosinophilic granuloma Intraosseous hemangioma Plasma-cell myeloma Giant-cell tumor Aneurysmal bone cyst Meningioma* Lymphoma* Metastasis*	Osteoma Osteosarcoma Meningioma* Lymphoma* Metastasis*	Meningioma* Hemangiopericytoma Lymphoma* Plasmacytoma* Metastasis*

*Metastasis, meninigioma, lymphoma, and plasmacytoma may be transdiploic, originating from the intracranial extraaxial soft-tissue, or intradiploic as primary bone lesions

## Pseudolesions

Cranial pseudolesions are very frequent and usually straightforward.

### Arachnoid (pacchionian) granulations

**Fig. 1:**
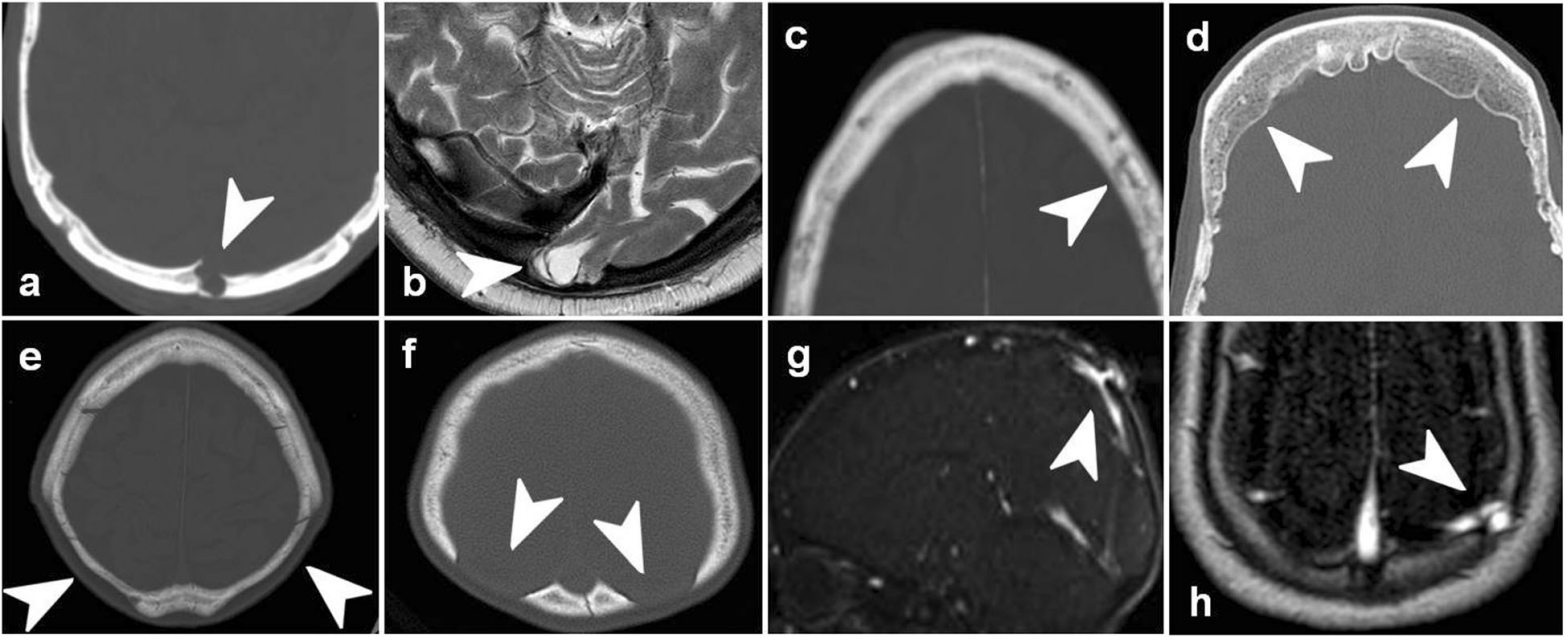
Calvarial pseudolesions. NECT (**a**, **c**–**f**), T2WI (**b**), MR venography (**g**, **h**). Parasagittal occipital arachnoid granulation (**a**). Occipital encephalocele (**b**). Dilated vascular canals and lacunae (**c**). Hyperostosis frontalis interna (**d**). Bilateral thinning of the parietal bones (**e**). Enlarged parietal foramina (**f**). Congenital sinus pericranii (**g**). Iatrogenic acquired sinus pericranii from a prior ventricular drainage (**h**)

Arachnoid (pacchionian) granulations (Fig. 1a) are the most common pseudolesion. They constitute normal protrusions of arachnoid into venous sinuses and diploe. They often grow and become hypertrophic with age. On imaging, they consist of non-enhancing, well-circumscribed bubbly filling defects in venous sinuses or lytic indentations through the inner table with a sclerotic rim when affecting the bone. CT and MRI signal corresponds to cerebrospinal fluid. They are usually located near the transverse or superior sinuses [5].

### Meningeal herniations

Meningeal herniations make up a continuum of disease involving progressive layers of central nervous system structures. Simple meningoceles have no relation to vascular structures and tend to appear in the sphenoid-wing, lamina cribosa, occipital region, and sphenoid-sinus walls. Meningeal herniations are common and usually increase with age due to bone weakening. They are often asymptomatic, but may cause intracranial hypotension or even seizures in the case of meningo-encephaloceles (Fig. 1b) [5]. Atretic cephaloceles are a type of congenital cephalocele containing dysplastic neuronal structures, normally occurring in the parietal midline and often accompanied by other midline-structure anomalies [6].

### Dilated vascular canals and lacunae

Dilated vascular canals and lacunae (Fig. 1c) are normal connections between dural and scalp veins that increase with age.

Awareness of these last three entities (Arachnoid granulations, Meningeal herniations and Dilated perivascular canals) is important because they often coexist in the elderly and may be misinterpreted as multiple lytic lesions, such as myeloma or metastases.

### Hyperostosis frontalis interna

Hyperostosis frontalis interna (Fig. 1d) is a benign process of unknown etiology, characterized by the thickening of the internal plate of the skull, which most commonly affects the frontal bone. It is much more common in women (16.4% females and 7% males) and correlates with age, obesity, parity, and diffuse idiopathic skeletal hyperostosis [7].

### Bilateral thinning of the parietal bones

Bilateral thinning of the parietal bones (Fig. 1e) is a rare idiopathic entity of unknown origin. This acquired slowly progressive anomaly found in middle-aged people is more frequently observed in women and is probably associated with osteopenia [8].

### Enlarged parietal foramina

Enlarged parietal foramina (Fig. 1f) are symmetric bilateral congenital bone defects in the parietal calvarium associated with gene deletions in chromosome 11p. They may be isolated or syndromic. If this is the case, they are associated with cortical dysplasia and vascular and skeletal anomalies [9, 10].

### Sinus pericranii

Sinus pericranii is an aberrant vascular connection between intracranial and extracranial veins through a bone defect. It is usually located near the dural sinuses and presents as a soft, palpable scalp mass. This abnormality can be congenital (Fig. 1g), in which case it is often obvious, but may also be acquired through trauma or iatrogenesis (Fig. 1h) and might be subtle in these cases. Identification is vital to avoid harmful biopsy [11].

## Predominantly lytic lesions

Lytic skull vault lesions encompass a wide range of diseases. We propose a systematic approach based on salient imaging and clinical characteristics (Table 2).

**Table 2:** Approach to lytic skull vault lesions

Young patient	Fibrous dysplasia (ground-glass matrix) Eosinophilic granuloma (“button sequestrum”)
Elderly and multiple	Metastasis Plasma-cell myeloma
Bone-expanding	Fibrous dysplasia (ground-glass matrix) Hemangioma (“spoke wheel”) Giant cell tumor (hypervascular, flow-voids) Aneurysmal bone cyst (hemosiderin levels)
Diffusion-restricting	Epidermal inclusion cyst (previous trauma) Dermoid cyst (fatty content)

### Fibrous dysplasia

Fibrous dysplasia (FD) accounts for 5 to 7% of benign bone tumors [12]. It is congenital, caused by a postzygotic mosaic mutation in the GNAS1 gene. This abnormality occurs and progresses throughout childhood and adolescence, and usually stabilizes in adulthood [13]. Most cases of FD are monostotic (75–80%), but craniofacial forms have a much higher tendency to being polyostotic [14–16]. Serum alkaline phosphatase may be occasionally elevated, but calcium, parathyroid hormone, and vitamin D levels are normal. A minority of polyostotic cases have associated syndromes such as McCune-Albright and Mazabraud syndromes. The former presents with *café-au-lait* spots and endocrine abnormalities including acromegalia and precocious puberty whereas Mazabraud syndrome presents as polyostotic FD with multiple soft-tissue myxomas [13, 17].

**Fig. 2:**
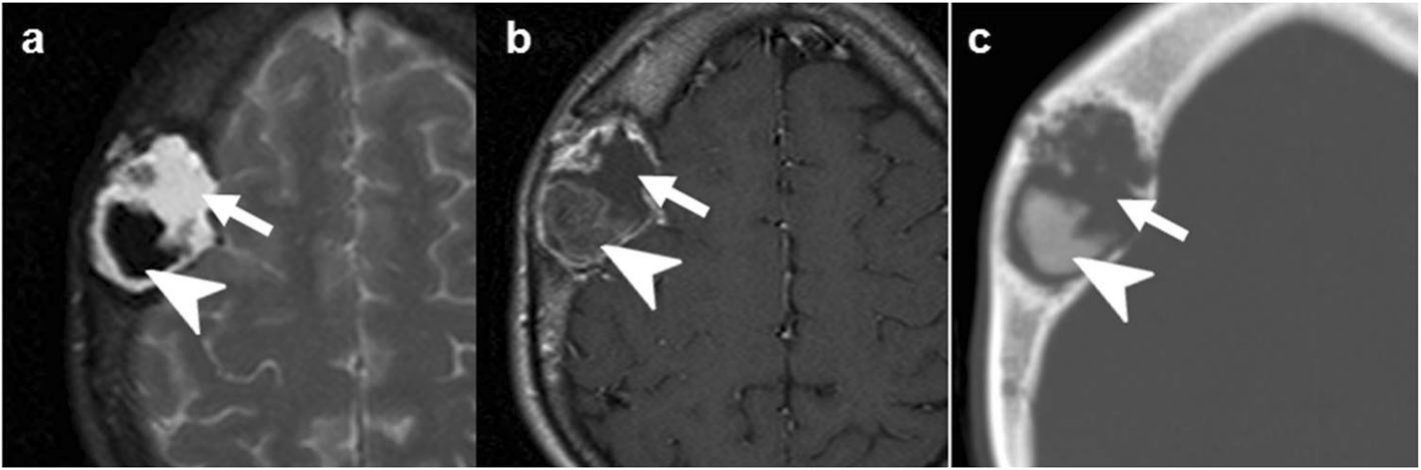
Fibrous dysplasia. T2WI (**a**), post-contrast T1WI (**b**), and NECT (**c**). Expansile lesion with distinct lytic-cystic component (arrows) and “ground-glass” fibrous tissue (arrowheads) clearly depicted on NECT, deeply T2-hypointense, which enhances heterogeneously

Radiologically, FD is a *focal*, well-defined bone-expanding lesion, which may be lytic, mixed, or sclerotic at the expense of a virtually pathognomonic “ground-glass” matrix. The prevalence of lytic components is greater in skull vault lesions so the “ground-glass” may be overlooked. On magnetic resonance imaging (MRI), the lytic components correspond to hyperintense areas on T2-weighted images (T2WI) and the “ground-glass” includes T2 hypointense fibrous tissue that enhances avidly. As a result, MRI can be misleading when interpreted without additional imaging (Fig. 2) [14, 15, 18].

### Epidermoid and dermoid cysts

Epidermoid cysts are slow-growing, non-neoplastic lesions. They may be congenital, deriving from ectodermal inclusion during neural tube closure or acquired secondarily to trauma or intervention. Despite their benign nature, expansive growth is possible [19, 20].

Dermoid cysts are mature teratomas lined with epithelium that may contain various mature tissue-types, typically located near the midline [20].

**Fig. 3:**
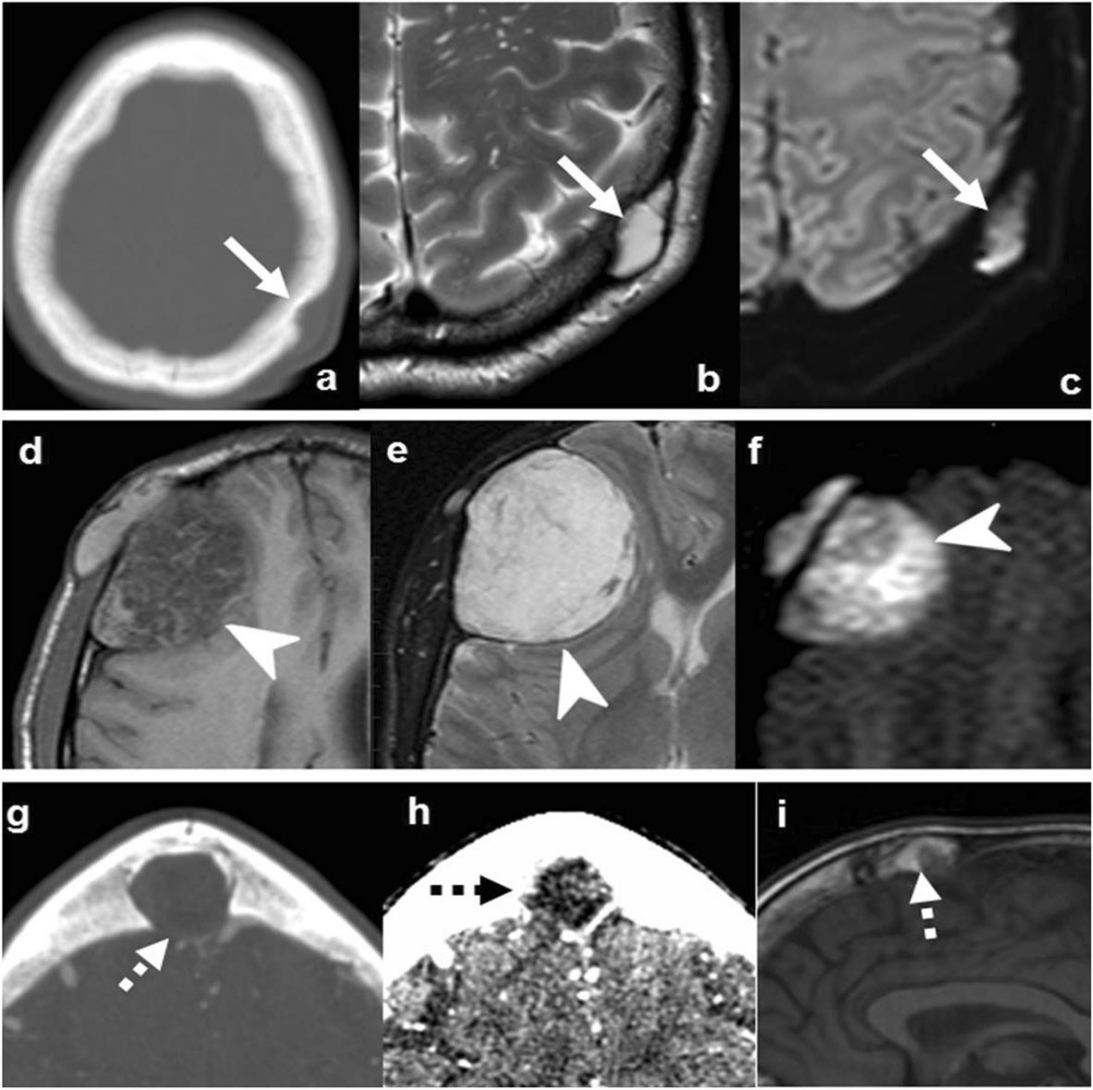
Epidermoid cysts (**a**–**c** and **d**–**f**) and dermoid cyst (**g**–**i**). Case 1 (**a**–**c**): Extracranial soft-tissue mass (arrows) remodeling the outer table on NECT (**a**), with fluid T2-signal (**b**), and markedly restricted diffusion (*b* = 1000) (**c**). Case 2 (**d**–**f**): Transdiploic mass (arrowheads), predominantly intracranial, T1WI (**d**) and T2WI (**e**) heterogenous and intense diffusion restriction (**f**). Case 3 (**g**–**i**). Frontal skull lesion (dashed arrows) disrupting the inner table on CT (**g**, **h**); content is CT-hypodense and T1-hyperintense, corresponding to fat (**i**)

On imaging, these lesions can arise from the bone itself or affect it by contiguity from the adjacent intra- or extracranial tissues. They are well-defined, expansile lesions which can cause adjacent non-aggressive bone lysis or remodeling. The cystic content of epidermoid can be heterogeneous with calcification and bleeding, but its features are often similar to fluid, with the most salient finding being intense diffusion restriction. Dermoids characteristically contain fat (Fig. 3) [19, 21].

### Eosinophilic granuloma

Eosinophilic granuloma is the focal bone presentation of Langerhans cell histiocytosis. It has been described in all ages, but mainly affects young infants and peak incidence occurs between 1 and 3 years of age. Annual incidence can reach 5.4 per million person-years [22]. This anomaly may affect any bone but has a special predilection for the skull. Prognosis is good and the majority of patients are cured [23]. Patients may suffer local pain and swelling [21, 24].

**Fig. 4:**
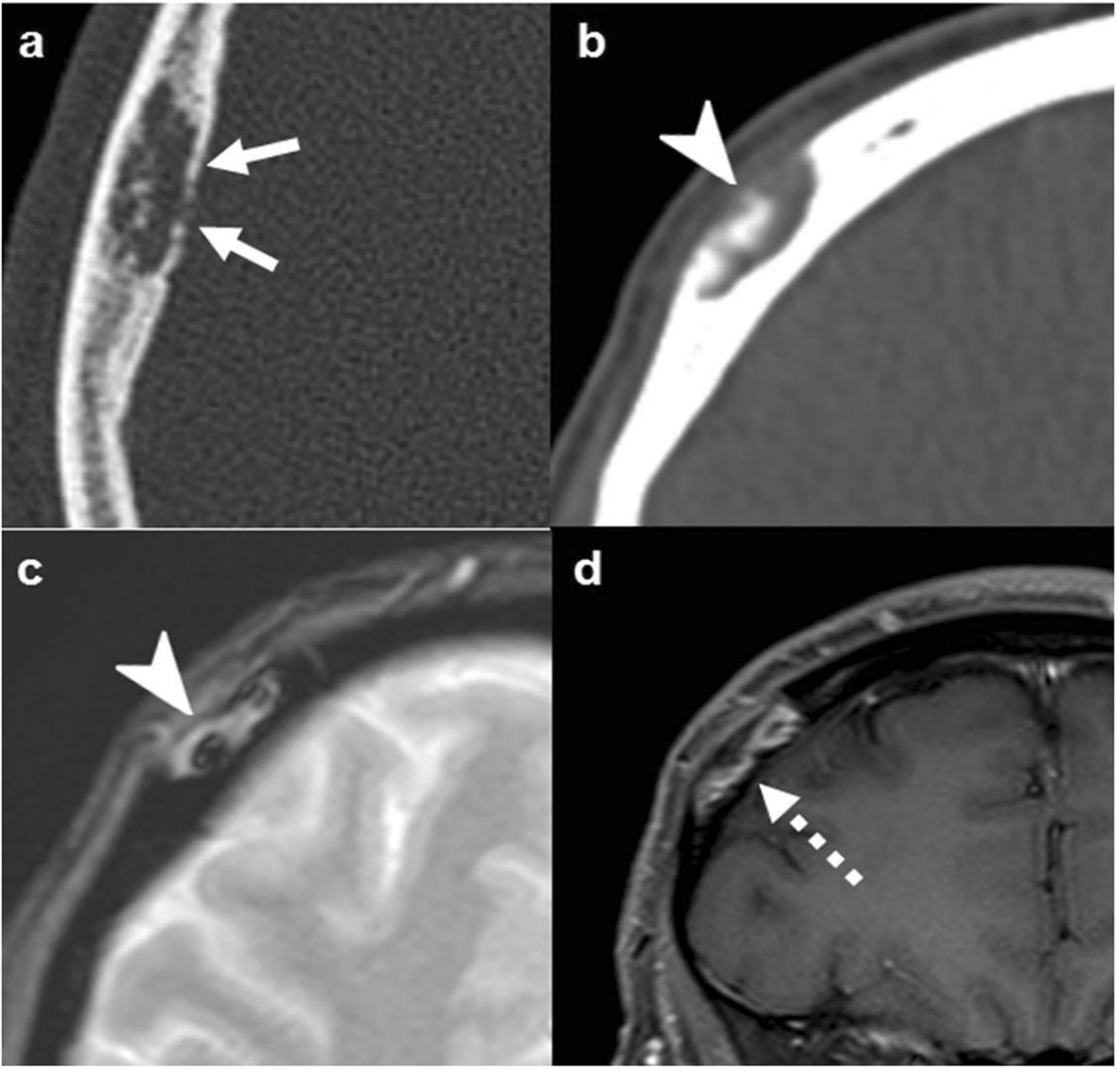
Eosinophilic granuloma. NECT depicts inner and outer table endosteal scalloping and erosions (arrows in **a**) and bone sequestra (arrowhead in **b**). On T2WI (**c**), the lesion is hyperintense and bone sequestra could also be suspected (arrowhead). T1WI post-contrast (**d**) demonstrates intense enhancement and cranio-caudal orientation (dashed arrow)

Skull lesions are typically well-defined and lytic, with sclerotic or non-sclerotic margins, and possible periosteal reaction. Endosteal scalloping (or erosions) and slight expansion beyond inner and outer tables determine the characteristic “beveled edges” and “hole within a hole” signs. A central “button sequestrum” is classically described. A tell-tale somewhat lenticular morphology, cranio-caudally oriented, may also be seen. MRI shows a T2-hyperintense lesion with variable degrees of contrast enhancement, and possible bone and soft tissue edema (Fig. 4) [21, 24].

### Intraosseous hemangioma

Intraosseous hemangioma accounts for 10% of benign neoplasms of the skull. It may occur at any age, though most commonly in the fourth to fifth decade, with a 3:1 female to male ratio [25]. Vertebrae are the most common location of hemangioma, followed by the calvarium. Symptomatic presentation is very rare, consisting of local pain, lump, or mass effect [26].

**Fig. 5:**
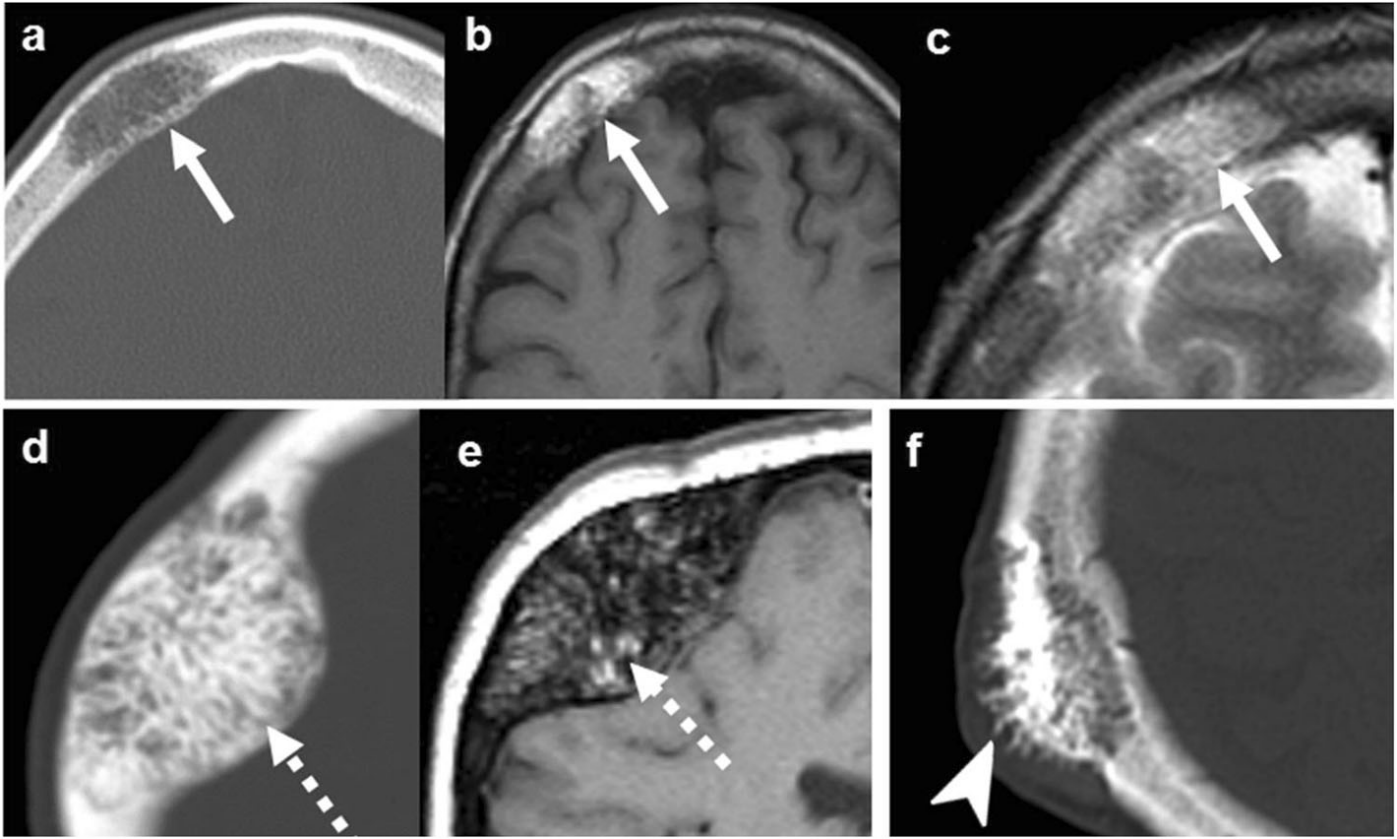
Bone hemangiomas. Case 1 (**a–c**): CT (**a**), T1WI (**b**), and T2WI (c) show well-defined lytic lesion, with thickened cortical and medullar trabecular spaces (arrows in **a**), and internal T1 and T2 hyperintense signal corresponding to fat and vascular tissue (arrows in **b** and **c**). Case 2 (**d**, **e**): CT (**d**) and T1WI (**e**) show marked bone expansion, fat content (dashed arrows), and strikingly enlarged trabecula producing a “spoke-wheel” appearance. Case 3 (**f**): CT depicts interruption of outer cortical table with overcoming enlarged spiculated trabecula simulating aggressive periosteal reaction (arrowhead)

Imaging is characteristic and almost always diagnostic. It consists of an intradiploic, circumscribed, lytic, and expansile lesion, which typically abuts the outer table. The classical appearance on CT consists of pronounced thickened trabeculae (“polka-dot” or “spoke-wheel”). MRI better demonstrates contrast enhancement and tissue characterization with internal T1 and T2 hyperintensities due to fat and vascular content, respectively [14]. Although rare, the enlarged bony trabeculae may grow beyond the cortical bone, producing a hair-on-end-like periosteal reaction or even dural or soft tissue contrast enhancing components, resembling a more aggressive or malignant lesion (Fig. 5) [26].

### Aneurysmal bone cysts

Aneurysmal bone cysts are benign lesions accounting for approximately 9% of benign bone tumors [12]. It can affect all age groups without gender predilection, but mainly occurs during the first two decades of life. Metaphyses of long tubular bones are the preferred location. The lesion can present with pain and eventually pathological fracture. It can be primary (arising de novo) or, in a few cases, secondary (associated in the skull with eosinophilic granuloma, fibrous dysplasia, osteoblastoma, or giant-cell tumor) [21, 27].

**Fig. 6:**
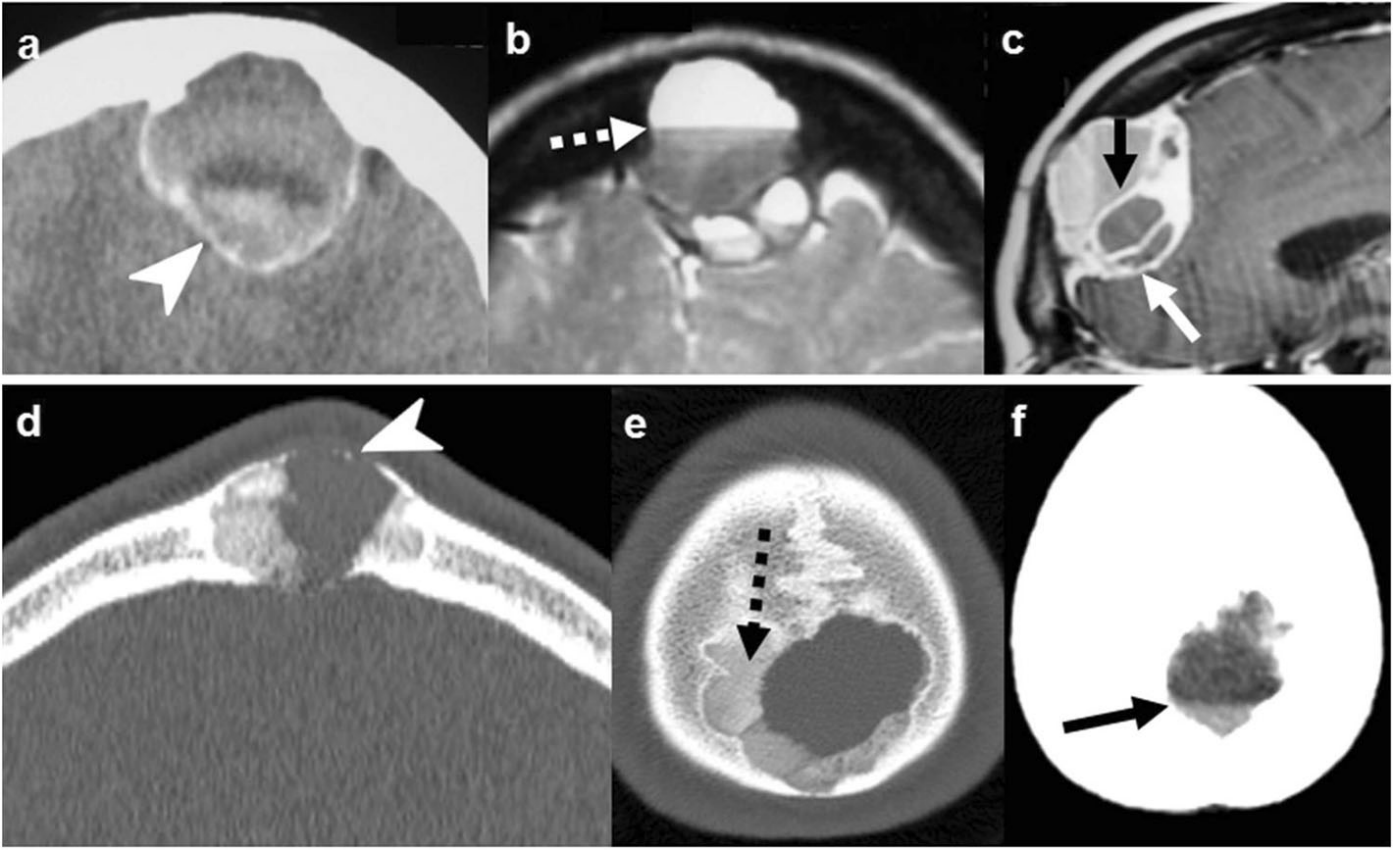
Aneurysmal bone cyst (ABC). Case 1 (**a**–**c**): Primary ABC: NECT (**a**), T2WI (**b**), and post-contrast T1WI (**c**) show an extremely expansile, lytic, multiloculated, septated lesion, with severe cortical thinning (arrowhead in **a**), fluid-fluid levels (dashed arrow in **b**), and lineal enhancement (arrows in **c**). Case 2 (**d**–**f**): Fibrous dysplasia with secondary ABC: NECT demonstrates an expansile, lytic lesion with severe cortical thinning (arrowhead in **d**), a characteristic fluid-fluid level (arrow in **f**), and associated areas of “ground-glass” matrix (dashed arrows in **e**)

Imaging demonstrates a focal, multilobulated, multicystic lesion with fluid-fluid levels and thin sclerotic margins. CT depicts bone-expansion and cortical thinning. The lesion is usually contained by periosteum, but in some cases, it may rupture or disappear. MRI highlights its multicystic components with the virtually pathognomonic fluid-fluid levels of hematic degradation by-products. The presence of non-cystic components suggests a secondary origin (Fig. 6) [21, 27].

### Giant cell tumor

Giant cell tumor accounts for 5% of all primary bone tumors and 20% of benign skeletal tumors, typically affecting young adults 20–45 years old [27]. It can present with pain or pathological fracture, mainly in long-bone epiphyses. This tumor is considered benign but may behave aggressively and recur [27, 28].

**Fig. 7:**
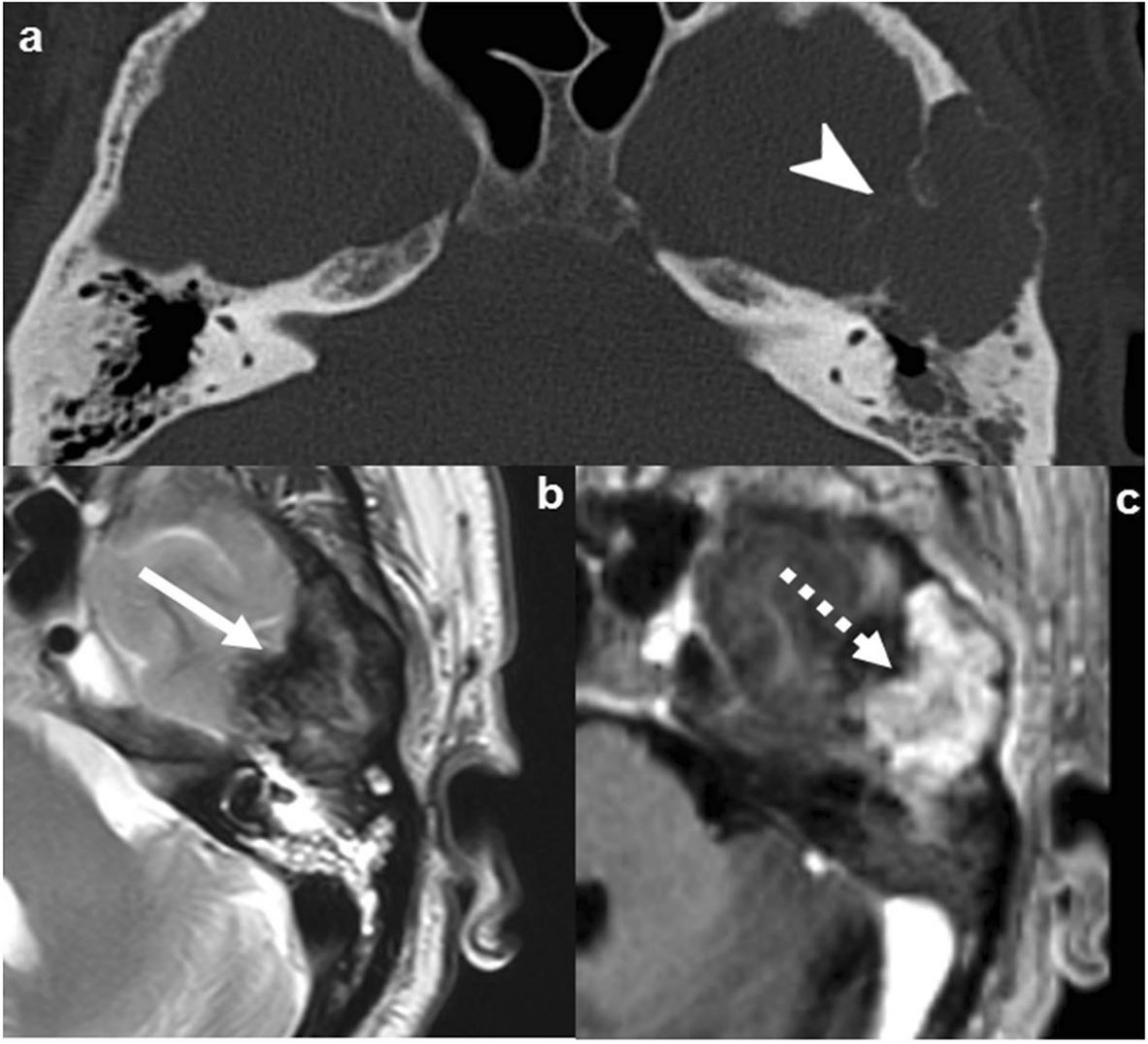
Giant cell tumor of the bone. Axial NECT (**a**), T2WI (**b**), and T1WI post-contrast (**c**). Purely lytic lesion with severe expansion and cortical thinning (arrowhead in **a**), markedly T2 hypointense tissue content (arrow in **b**), and avid contrast-enhancement (dashed arrow in **c**)

Imaging shows a solid or mixed solid-cystic mass with bone expansion, prominent cortical thinning (often partially broken or disappeared), and well-defined but non-sclerotic margins. Solid components tend to be NECT hyperdense and T2 hypointense attributed to hypercellularity, hemorrhage, or fibrous matrix. The tumor is highly vascularized and shows intense contrast enhancement. An associated secondary ABC should be suspected when cystic areas are found (Fig. 7) [27].

### Metastases

Metastases are the most prevalent neoplastic bone lesions. They are especially relevant in breast and prostate cancers, in which there is a prevalence of circa 70% in postmortem studies [29]. The presenting symptoms range widely from asymptomatic to serious complications, and prior cancer history is frequent [30].

**Fig. 8:**
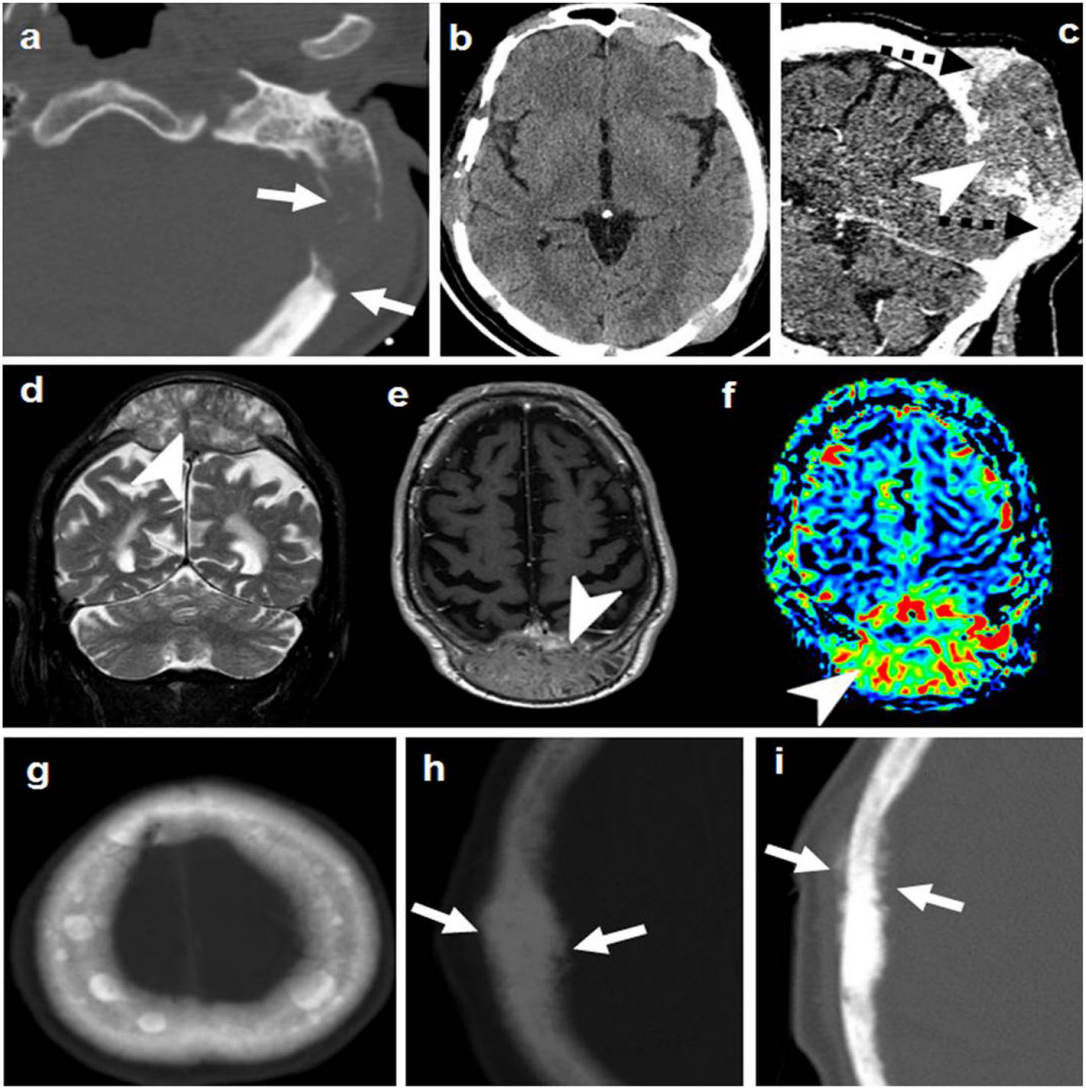
Metastases. Lytic metastases (**a**–**f**). CT of three patients (**a**–**c**) shows: **a** Solitary with narrow zone of transition (arrows), **b** multiple with slight soft-tissue components, and (**c**) solitary, giant, with mixed necrotic (arrowhead) and hypervascular (dashed arrows) soft tissue. Another case (**d**–**f**) with T2WI, T1WI post-contrast, and DSC-PWI shows solitary, giant, solid-enhancing, and hypervascular soft-tissue component (arrowheads). **g**–**i** Three cases of blastic metastases. Multiple (**g**). Aggressive periosteal reaction (arrows in **h**–**i**)

The imaging pattern of bone involvement is very variable. The lesions are usually focal, lytic (with varying degrees of bone destruction and soft-tissue components), narrow zones of transition, and periosteal reaction. Expanding lytic (or blowout) metastases are typical of renal-cell or thyroid papillary carcinomas. Permeative patterns of bone destruction with possible disproportionate soft-tissue components may be observed in highly cellular small-round cell tumors that can grow through the Haversian canals (PNET, small-cell lung cancer). Hypervascular tumors such as renal-cell, thyroid cancer, melanoma, and hepatocarcinoma may present avidly contrast enhancing and highly perfused metastases. Finally, prostate and breast tumors are the usual suspects with blastic metastases (Fig. 8) [31].

### Myeloma

Myeloma is a hematological disorder that generally affects elderly patients (median age 68–70), rarely those under 40. It accounts for 1–2% of all cancers and around 17% of hematologic malignancies [32], with an annual incidence of 4–5 cases per 100,000 [33]. It most frequently presents with multifocal pain and has a poor prognosis [34].

The most common and well-known imaging pattern of bone involvement in myeloma is multiple punched-out lytic lesions. However, the disease can also present with diffuse osteopenia-like medullary bone-infiltration; exceptionally, and exclusively associated with POEMS syndrome, the lesions can be sclerotic.

On CT the lesions are slightly dense intrinsically. MRI demonstrates intermediate signal and often markedly restricted diffusion, with avid contrast-enhancement (Fig. 8) [34].

The axiom that “multiple aggressive lytic lesions in the elderly patient are myeloma or metastases until proven otherwise” is a well-known medical postulate. Some authors add that multiple metastases rarely affect mandible, in contrast to multiple myeloma.

### Plasmacytoma

**Fig. 9:**
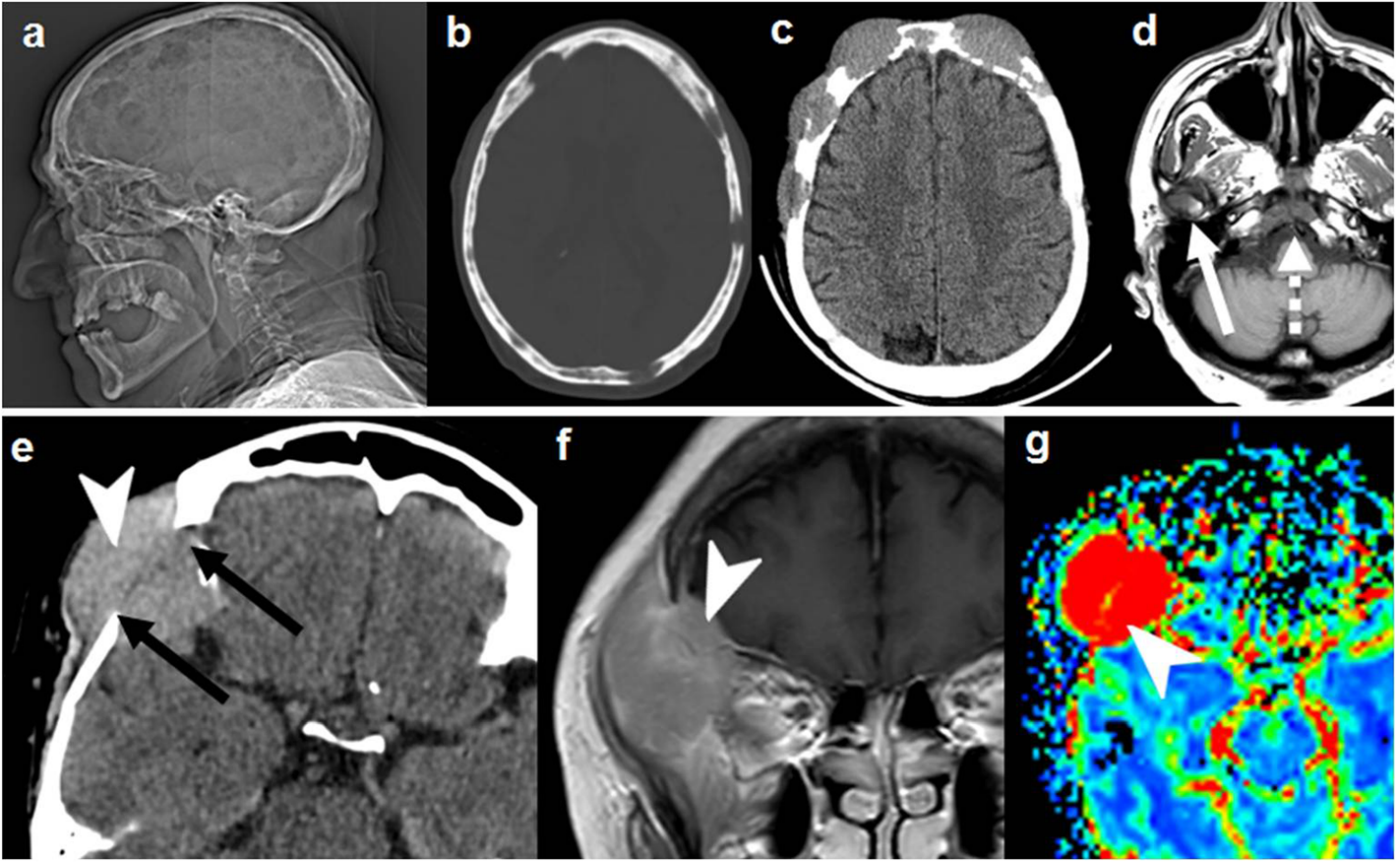
Myeloma and plasmacytoma. Upper row. Myeloma. X-ray (**a**) NECT (**b**, **c**) and T1WI (**d**). Multiple “punched-out” lytic lesions with variable soft tissue components (**a**–**c**). Mandibular lesion suggesting myeloma instead of metastases (arrow in **d**). Note also the clival lesion (dashed arrow in **d**). Lower row. Plasmacytoma. NECT (**e**), T1WI post-contrast (**f**), and DSC-PWI (**g**). Single purely lytic transdiploic lesion with narrow zone of transition (arrows in **e**), hyperdense, hyperenhancing, and hypervascular (arrowheads respectively in **e**–**g**)

Plasmacytoma is the least common solitary counterpart of myeloma, and the prognosis is slightly better [34]. It usually appears as a purely lytic lesion which surpasses the cortical margins with soft-tissue components, restricts diffusion, and is hypervascular (Fig. 9).

## Predominantly sclerotic lesions

The list of sclerotic calvarial lesions is more limited than the lytic ones: osteoma, blastic metastases, and the infrequent osteosarcoma (as well as the exceptional POEMS-associated myeloma). As previously noted, some other lesions such as fibrous dysplasia and hemangioma can also show somewhat sclerotic components. Lymphoma often causes bone sclerosis but will be treated separately.

### Osteoma

Osteoma is a benign, slow-growing lesion. Osteomas in the cranial vault are mainly asymptomatic, while sinonasal ones can lead to sinus obstruction and sinusitis. Gardner syndrome is associated with multiple osteomas along with familial polyposis, fibromatosis, and dental and skin lesions [28].

**Fig. 10:**
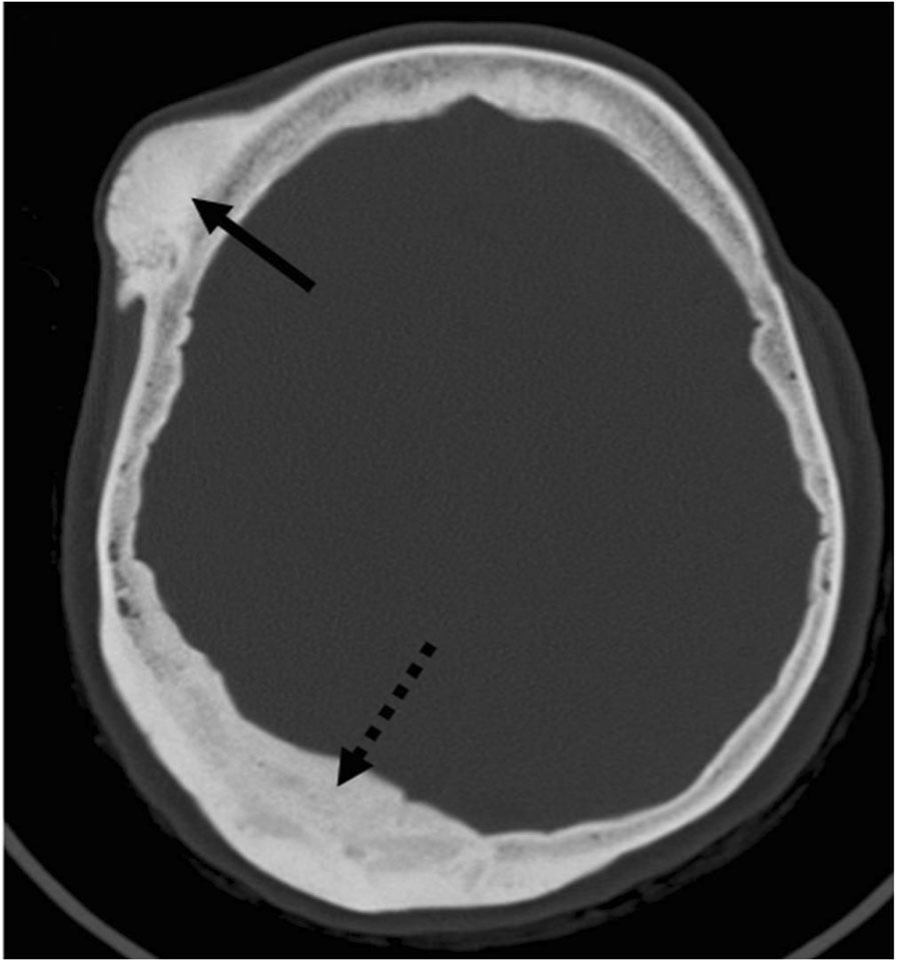
Osteomas. Multiple in Gardner syndrome. NECT show mass-like proliferation of normal appearing cortical bone in the frontal (arrow) and cortical and medullary bones in the parieto-occipital (dashed arrow) lesions

Imaging is evident and diagnostic, identifying exophytic, rounded, sclerotic lesions attached to the bones. On the basis of their composition, they may be ivory (cortical bone alone), mature (normal bone with peripheral cortex and central marrow), or mixed (with cortical and marrow heterogeneously distributed) (Fig. 10) [35].

### Blastic metastases

Blastic metastases are less common than lytic ones. They should be considered in the differential of a sclerotic, aggressive, lesion in adults, especially when there is a history of cancer, associated symptoms, or recent appearance. Prostate and breast tumors have been classically associated with blastic metastases, but neuroendocrine tumors, urothelial carcinomas, and small-round cell tumors should also be considered (Fig. 8).

### Osteosarcoma

Osteosarcoma, accounting for only 1% of cancers diagnosed in the USA, is the most common primary malignant tumor of the bone, with a bimodal age distribution having a first peak at 10–14 years of age and a second over 40 [36]. It presents as a quick-growing painful mass. Prognosis is poor but greatly depends on the surgical resection and presence of metastasis [37, 38].

There are several histological subtypes, but the most common (82%) is the osteoblastic, which presents as a highly destructive, aggressive blastic lesion, with periosteal reaction, a soft tissue component, and very specific “cloud-like” osteoid matrix [28, 38].

**Fig. 11:**
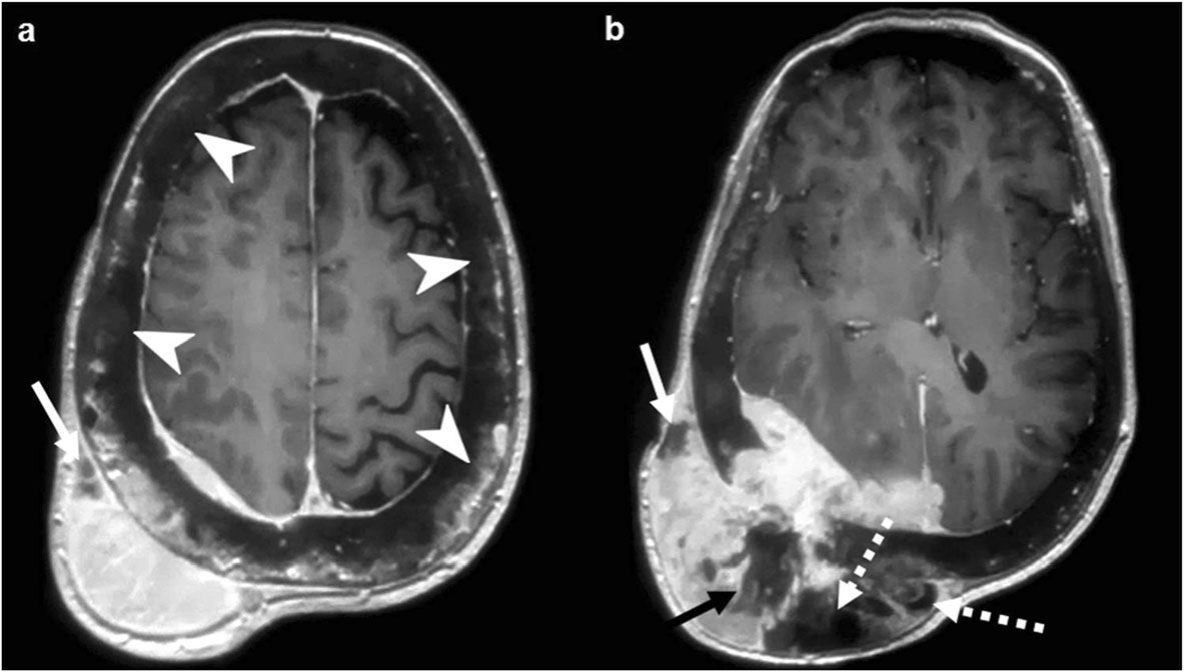
Osteosarcoma. Secondary, “pagetic.” T1WI post-contrast (**a** and **b**). Aggressive, destructive, giant, and transdiploic lesion with extensive intra- and extracranial soft-tissue component containing iso to hypointense areas of probable necrosis (arrows in **a** and **b**) and deeply hypointense foci of suspected bone matrix (dashed arrows in **b**). Note the underlying abnormal calvarial bone with diffuse expansion, cortical thickening and heterogeneous multifocal variable signal intensity, and enhancement (arrowheads) consistent with Paget disease

Pediatric cases are almost all primary, whilst adult cases are often secondary, arising from pagetic or irradiated bone [36]. Primary osteosarcoma of the skull vault in adults is exceptional and limited to a few case reports. Therefore, when it is suspected, it is probably secondary, and “pagetic” or irradiated bone nearby should be sought (Fig. 11) [39].

## Transdiploic lesions

This category has been added separately to include mainly dural-based lesions, which can potentially involve and cross the calvarium. The most paradigmatic example would be meningioma. However, several other entities with different histology, management, and prognosis may closely mimic this behavior, such as hemangiopericytoma, lymphoma, metastasis, and plasmacytoma.

### Meningioma

Meningioma is the most common cranial tumor, accounting for 33.8% of all primary brain and CNS tumors, with a (2.3:1) female predilection, and a median age at diagnosis of 65 years [40, 41]. Intradiploic and primary extraosseous meningiomas, however, account for less than 2% of all meningiomas. Neurofibromatosis type 2 and schwannomatosis predispose to multiple meningiomas [41, 42].

There are different grades of malignancy and prognosis not attached to imaging features. As a rule, there is an avidly and homogenously enhancing extraaxial mass with “dural-tails,” calcifications, and fairly specific hyperostosis of the adjacent bone. Another classic trait is a great tendency to infiltrate contiguous venous sinuses.

**Fig. 12:**
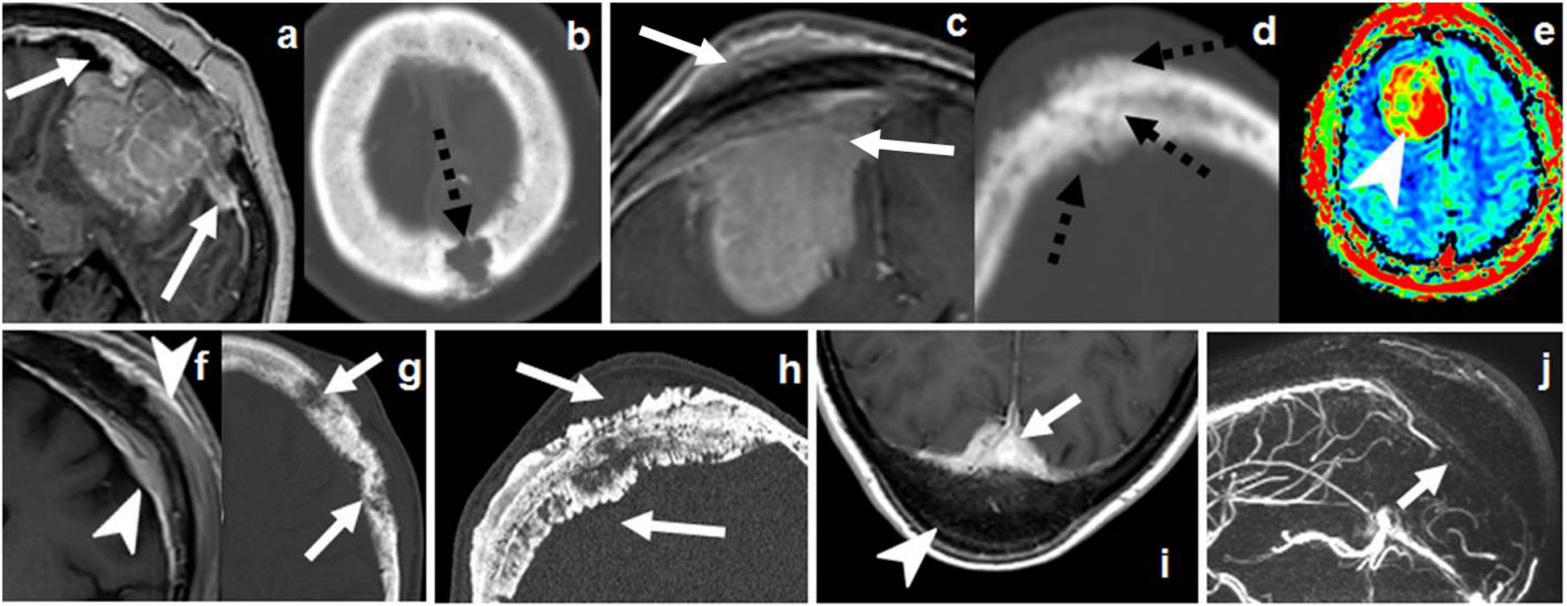
Meningioma. Great polymorphism. Case 1 (**a**, **b**): Post-contrast T1WI (**a**) and CT (**b**): dural-based with dural tails (arrows) and bone lysis (dashed arrows). Case 2 (**c**–**e**): Post-contrast T1WI (**c**), CT (**d**), and DSC-PWI (**e**): dural-based hypervascular (arrowhead) with transdiploic growing (arrows), bone sclerosis-permeation, and aggressive-like periosteal reaction (dashed arrows). Case 3 (**f**, **g**): Post-contrast T1WI (**f**) and CT (**g**): diploic-centered plaque-like with intra- and extracranial laminar soft-tissue (arrowheads) and bone lysis-permeation (arrows). Case 4 (**h**): CT shows diploic-centered lesion with great spiculated hyperostosis (arrows). Case 5 (**i**, **j**) Post-contrast T1WI (**i**) and MR venography (**j**): diploic-centered with great homogeneous smooth hyperostosis (arrowhead) and intracranial soft-tissues characteristically invading the superior longitudinal venous sinus (arrows)

Nevertheless, it is very polymorphic on imaging, and bone invasion can take different forms instead of the specific hyperostosis: well-defined lytic (simulating metastases or plasmacytoma), permeative/moth eaten (simulating lymphoma or small-round cell metastasis), or even aggressive periosteal reaction (eventually misleading to the suspicion of osteosarcoma or metastasis) (Fig. 12) [18, 43, 44].

In this sense, functional imaging techniques may help in the characterization of meningiomas with atypical patterns of bone involvement. Proton magnetic resonance spectroscopy (MRS) centered in the soft-tissue component may demonstrate increased choline (Cho, 3.21 ppm), decreased creatine (Cr, 3.03 ppm), elevated glutamine/glutamate (Glx, 2.0–2.05 ppm), and, characteristically, alanine (Ala, 1.47 ppm doublet inversion on long echo times). *N*-acetil-aspartate (NAA, 2.02) in extraaxial tumors is absent or very low, as it is a marker of neuronal tissue [43, 45]. MR T2* dynamic susceptibility contrast-perfusion-weighted imaging (DSC-PWI) time-intensity curves (TIC) within a region of interest (ROI) in the soft-tissue component typically shows a curve with little or no return to baseline. Very high values of relative cerebral blood volume (rCBV) are also a characteristic feature (Fig. 15) [43, 46, 47].

Overexpression of somatostatin receptors in meningiomas makes them a target for their PET ligands, such as ^68^Ga-DOTATOC, ^68^Ga-DOTATATE, and ^68^Ga-DOTANOC. Uptake of these ligands is highly sensitive for meningiomas, but also present and used for imaging of neuroendocrine tumor metastasis [48]. ^68^Ga-DOTATATE maximum standardized uptake value (SUVmax) has also proven to be a predictor of tumor growth rate and trans-osseous expansion. These properties are expanding the potential use of this ligand for detection and differential diagnosis, grading, treatment planning, and treatment monitoring of meningiomas [48].

### Hemangiopericytoma

Hemangiopericytoma (or solitary fibrous tumor) is an uncommon, highly vascular, dural-based tumor that affects adults, accounting for less than 1% of all CNS tumors with a median age of 40–60 years with a slight male predilection. There are different grades of malignancy and prognosis [49, 50]. Hemangiopericytoma and solitary fibrous tumor were previously considered two separate entities but have been combined as one single entity in the latest 2016 WHO classification of central nervous system tumors [49].

**Fig. 13:**
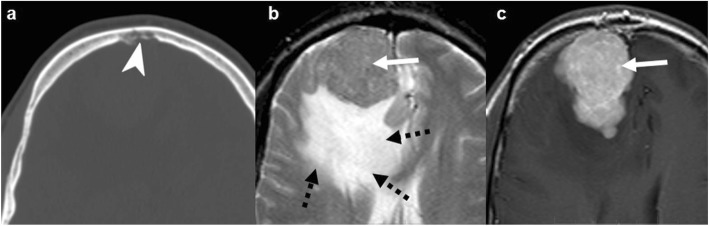
Hemangiopericytoma. CT (**a**), T2WI (**b**), and post-contrast T1WI (**c**). Dural-based “mushroom-like” mass (arrows) with brain edema (dashed-arrows), intense homogeneous enhancement, and foci of bone lysis (arrowhead)

On imaging, it presents as an extra-axial, avidly enhancing mass, with flow voids and possible “dural tail.” This appearance makes it difficult to differentiate it from the much more common meningioma. However, unlike the latter, hemangiopericytoma does not calcify and does not present hyperostosis since bone involvement is purely lytic. When the dural-base is narrow, it shows the classical “mushroom” appearance (Fig. 13) [51].

MRS does not present the characteristic alanine of meningiomas and very specifically demonstrates high myoinositol (Myo, 3.55 ppm) (Fig. 15) [52].

### Lymphoma

Lymphoma can affect all ages, with an incidence peak between 50 and 60 years of age, while it is exceptional under 10 years of age. Primary bone lymphoma accounts for 7% of all bone malignancies and 5% of all extranodal lymphomas [53].

**Fig. 14:**
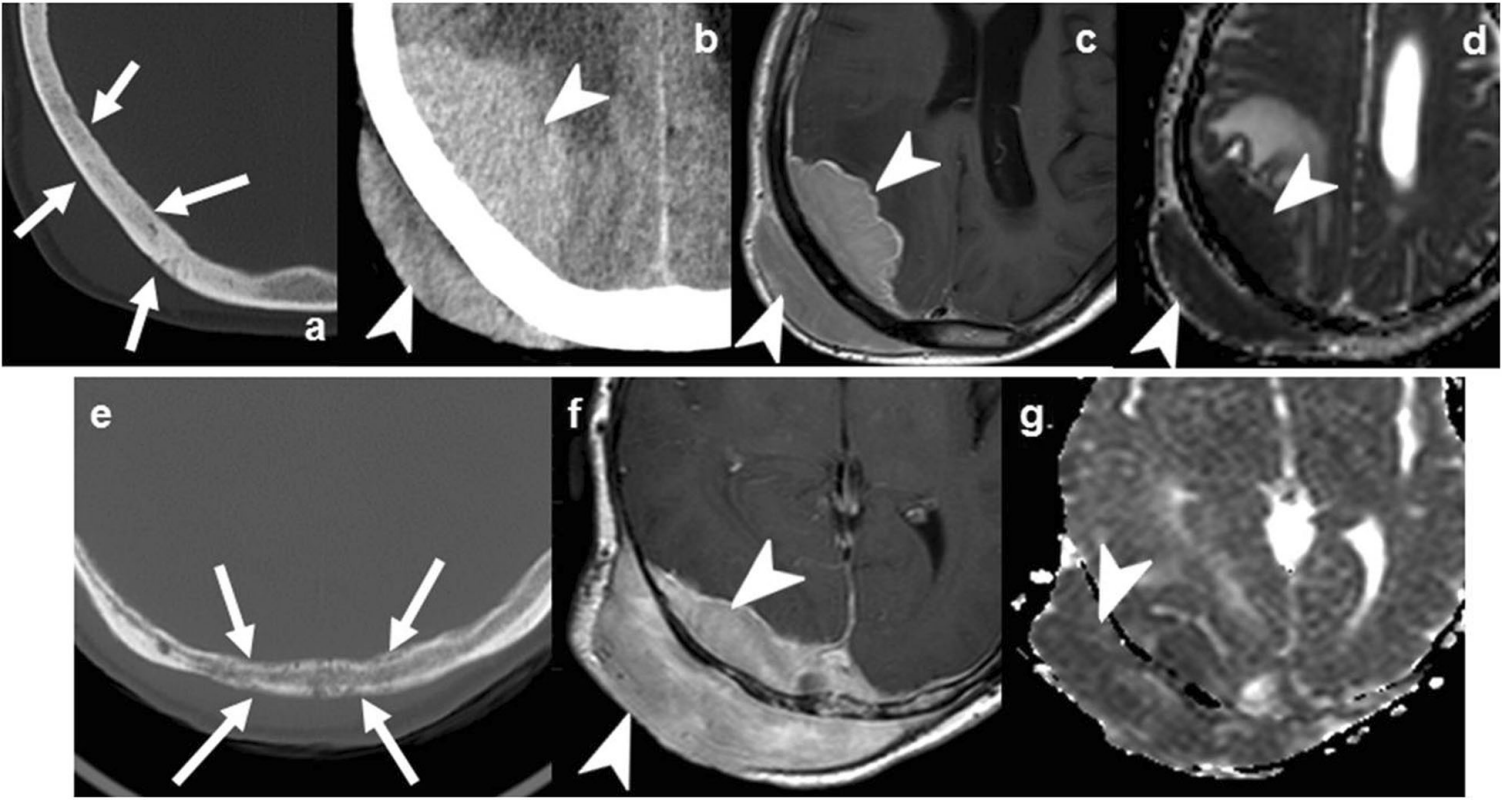
Lymphoma. Case 1 (**a**–**d**): NECT (**a**, **b**), post-contrast T1WI (**c**), and ADC map (**d**). Transdiploic mass with bone subtle sclerosis (arrows) and disproportionate soft-tissue components dense on CT, homogenously enhancing, and diffusion-restricting (arrowheads). Case 2 (**e**–**g**): Similar findings with bone lytic-permeation (arrows) instead

Imaging characteristics of calvarial lymphoma are those of small-round cell tumors. It can be lytic, sclerotic, or mixed, but most specifically permeative with transdiploic spread through the Haversian canals, with little or no bone destruction and with a disproportionate soft tissue component [54]. Soft tissue component tends to be NECT hyperdense, T2WI hypointense, and highly diffusion-restricting, due to hypercellularity. Contrast-enhancement is intense and homogeneous (Fig. 14) [18].

**Fig. 15:**
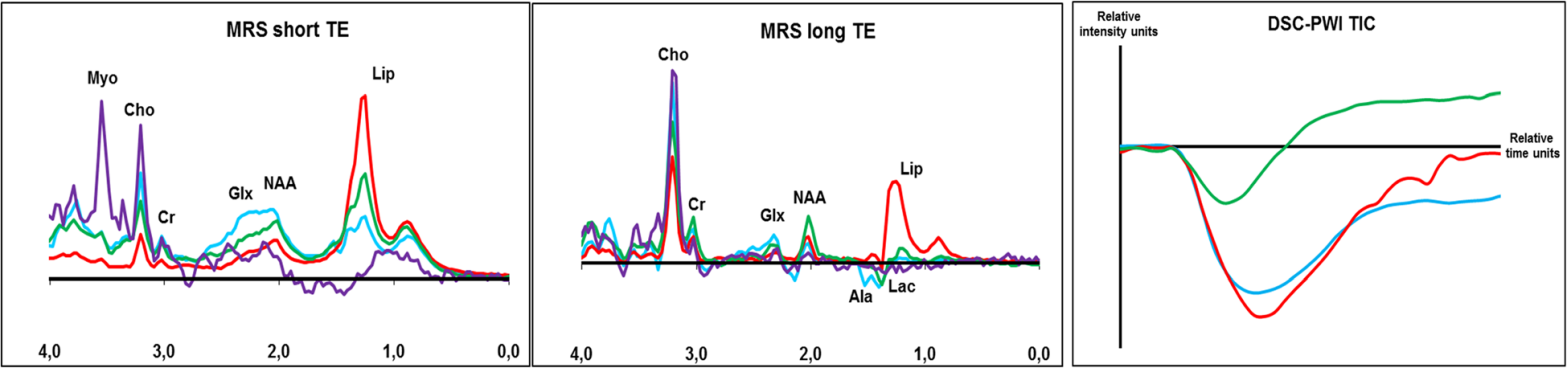
Characteristic MR spectroscopies and DSC-PWI TIC of transdiploic lesions. Green: lymphoma. Spectroscopy: high Cho + lip-lac. DSC-PWI TIC: recovers fast and ends far above the baseline. Blue: meningioma. Spectroscopy: high Cho, high Glx, Ala. DSC-PWI TIC: recovers slow and remains far under the baseline. Purple: hemangiopericytoma. Spectroscopy: high Myo. Red: metastasis. Spectroscopy: high lip. DSC-PWI TIC: recovers slowly and remains slightly under the baseline

MRS evidences high choline, some lipids (Lip, 0.9 and 1.3 ppm), and lactate (Lac, 1.31 ppm doublet inversion on long echo times) [55]. DSC-PWI TIC characteristically shows a curve that clearly ends above the baseline, with intermediate rCBV (Fig. 15).

### Metastases and plasmacytoma

Metastases and plasmacytoma can present as single transdiploic lesions. A large amount of necrosis in the soft tissues is quite typical of metastases but uncommon in meningioma/hemangiopericytoma, lymphoma, or plasmacytoma. The diagnostic dilemma encompasses metastases when solid and hypervascular.

Metastasis MRS demonstrates strikingly high lipids attributable to necrosis. Plasmacytoma, with scarce evidence, may expose high choline with some lipids or lactate, resembling lymphoma. Metastasis DSC-PWI TIC typically exposes a curve with slowly progressive slight or no return to the base line and variable rCBV, usually notably high.

### Summary of functional imaging techniques that may provide a clue when facing aggressive transdiploic lesions with soft tissue component

-Lymphoma (and less widely assumed, plasmacytoma) characteristically presents marked diffusion restriction.

-MRS evidences virtually pathognomonic alanine in meningioma, high myoinositol in hemangiopericytoma, strikingly high choline in lymphoma (and plasmacytoma), and high lipids in metastasis [43, 45, 46, 52]. MR DSC-PWI TIC has a very specific pattern in lymphomas with a curve that recovers and ends above the baseline, while meningioma and metastasis, in contrast, show a TIC that stays under the baseline [55, 56].

-PET imaging with ^68^Ga-DOTATATE is highly sensitive for meningiomas but can also be seen in neuroendocrine tumor metastases. SUVmax correlates with cell growth and tumor invasiveness and may be of used for diagnosis, grading, and follow-up [43].

Concepts presented in Table 3 and Fig. 15.

**Table 3:** Useful MR spectroscopy and MR perfusion criteria for transdiploic lesions

	MR spectroscopy	MR perfusion
Meningioma	Alanine (1.47 ppm): specific ↑Glutamate+Glutamine ↑Cho/Cr ratio	↑↑↑ rCBV (hipervascular) Slow-progressive return to baseline
Hemangiopericytoma	Myoinositol: Specific No alanine ↑ Cho/Cr ratio	Similar to Meningioma (hypervascular)
Lymphoma/small-blue round-cell	↑↑↑ Choline +/− Lipids or lactate	Recovery curve ABOVE baseline Slightly ↑ CBV (ratio 1–1.5)
Metastasis	↑↑ Lipids (tumor necrosis)	Recovery curve BELOW baseline

### Miscellany: metabolic, inflammatory infectious, and systemic disease

**Fig. 16:**
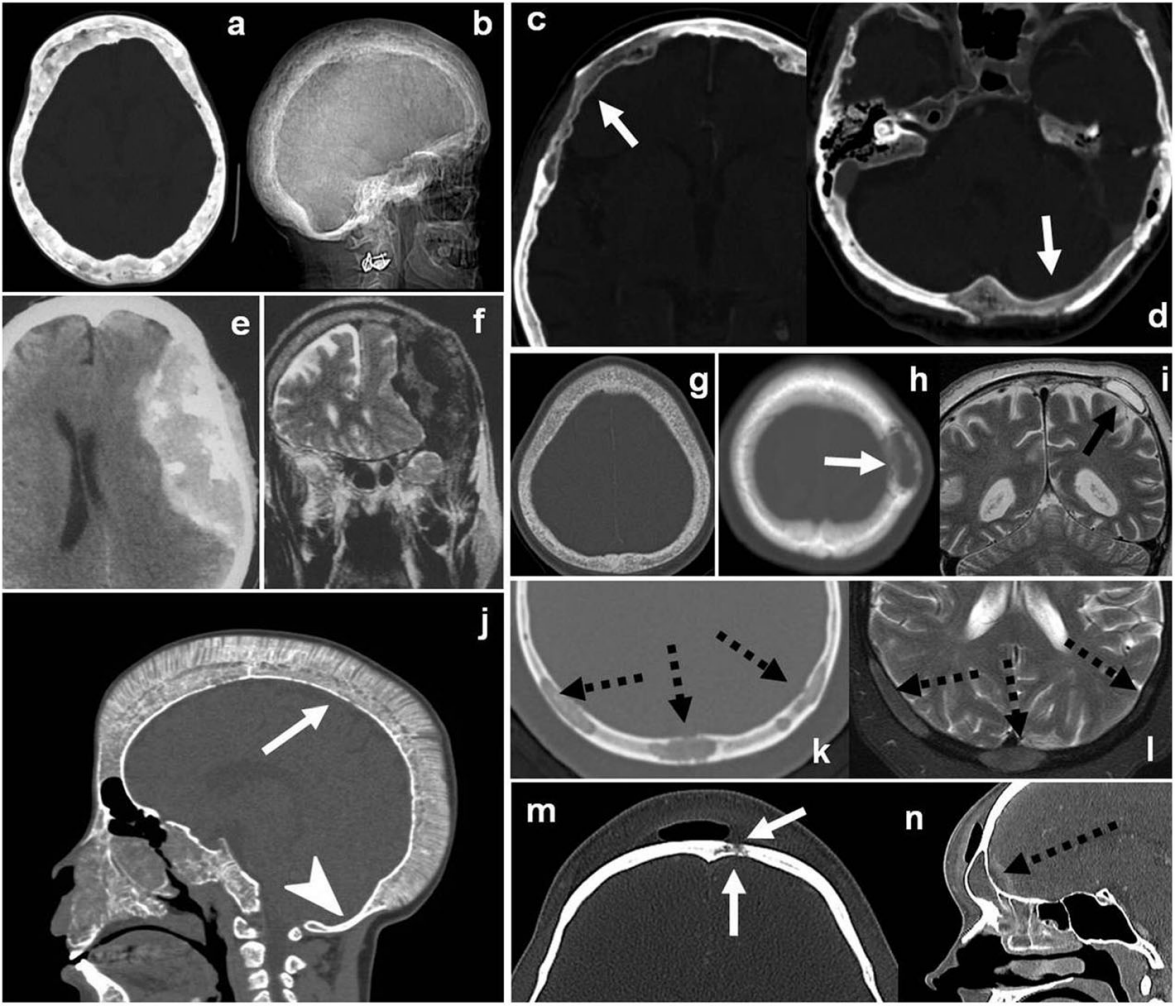
Miscellany. Paget disease (**a**, **b**) CT (**a**) and X-ray (**b**): Characteristic mixed bone lysis and sclerosis, cortical bone thickening, and expansion. Osteoporosis circumscripta cranii CT (**c**, **d**): large geographic radiolucent areas involving medullar and cortical bone in frontal and occipital regions (arrows). Amyloidoma (**e**, **f**) CT (**e**) and T2WI (**f**): Giant heterogeneous mass with marked T2 hypointensity and calcifications. Renal osteodystrophy CT (**g**): shows characteristic “salt-and-pepper pattern.” Brown tumor (**h**, **i**): unspecific well-defined, cystic appearance (arrows). Thalassemia CT (**j**): Diffuse diploic widening and “hair-on-end” appearance (arrow) with characteristic occipital bone preservation (arrowhead). Bone sarcoidosis (**k**, **l**) CT (**k**) and T2WI (**l**): Mixed predominantly lytic multiple lesions with lace-like internal pattern of calcification and T2 hypointensity (dashed arrows). Osteitis CT (**m**, **n**): Bone focal lysis and erosions of osteitis (arrows) contiguous to a frontal sinusitis complicated with intracranial laminar abscess (dashed arrow)

Finally, we must mention some frequent and anecdotal entities that may be encountered in the skull (Fig. 16).

### Paget’s disease of bone

Paget’s disease of the bone is very frequent in the elderly with a slight male predilection, with a reported incidence of 5/10,000 person-years among men and 3/10,000 in women 75 years of age, and a prevalence of 2.5% in men and 1.6 in women over 55 years of age [57]. It can present as inflammation and hyperemia. Imaging is characterized by medullary and cortical bone expansion and thickening, as well as coexistence of lytic and sclerotic lesions in variable degree depending on the phase of the disease (osteoclastic vs osteoblastic). Osteoporosis circumscripta cranii is considered an early stage (lytic phase) of the disease with a characteristic imaging pattern consisting of geographic, large, patched, radiolucent areas involving both medullar and cortical bone, and usually located in frontal and occipital regions [58].

### Renal osteodystrophy

Renal osteodystrophy appears as a diffuse “salt-and-pepper” pattern, and Brown tumor as a non-specific focal lesion in patients with chronic renal failure [59].

### Sarcoidosis

Sarcoidosis bone involvement is uncommon and predominates in the hands and feet. It typically presents as unspecific lytic lesions, which may disrupt the bone cortex and show an internal “lace-like” pattern of calcification [60].

### Thalassemia

Thalassemia is a hemoglobinopathy that runs its course with various well-known bone manifestations. The “hair-on-end skull” is very characteristic, with preservation of the non-hematopoietic occipital bone.

### Osteopetrosis

Osteopetrosis is an uncommon hereditary disorder that results from defective osteoclasts. Bones become sclerotic and thick while weak and brittle. There are many classifications, but the classically known as “benign” or “adult” osteopetrosis (an autosomal dominant variant) is the less severe form of osteopetrosis. A subtype of this entity (often known as type 1) usually affects the cranial vault with diffuse thickening and sclerosis resulting in typical appearance of “bone within a bone.” Clinically, it often presents with cranial nerve compressive palsies [61].

### Osteitis and osteomyelitis

Osteitis and osteomyelitis are usually related to paranasal sinus infections, facilitating diagnosis.

### Amyloidosis

Anecdotally, amyloidosis can affect the skull vault in the form of amyloidoma. The main learning points of the review are summarized in Table 4.

**Table 4:** Learning points

Pseudolesions: Very common. Know-them to recognize them	
Meningioma and hemangioma can simulate aggressive periosteal reaction	
Meningioma: Calcification, dural tail, intense enhancement, hyperostosis. Beware of variable bone involvement. 1H-MRS: Alanine (specific)	
Hemangioma: Expansile, trabeculated, spoke-wheel pattern, fatty content.	
Epidermal inclusion cyst: Previous injury, DWI restriction	
Dermoid cyst: DWI restriction, fatty content	
Fibrous dysplasia: Young patient. “Ground-glass” matrix virtually pathognomonic. Possible predominant lytic-cystic component in calvarium	
Eosinophilic granuloma: Young patient with focal lytic lesion with “button sequestrum”	
Giant-cell tumor: Hypervascular bone expansion lesion with flow-voids	
Aneurysmal bone-cyst: Bone-expanding lesion with fluid hemosiderin levels. May be secondary if prominent solid components	
Myeloma/metastastasis: Elderly patients with multiple lesions	
Blastic metastasis: Prostate, breast, transitional cell, neuroendocrine, PNET, lymphoma	
Hypervascular metastasis: thyroid, renal, hepatocarcinoma, neuroendocrine and melanoma	
Transdiploic or aggressive single lesion: Differential of plasmacytoma, metastasis, lymphoma or meningeal lesion (meningioma/ SFT). Functional imaging can be useful in narrowing differential	

## Conclusions

The skull vault can be affected by a wide variety of entities that present differently in this location compared to other parts of the human anatomy. A systematic approach based on key CT and MRI findings may be useful during the diagnostic process in order to avoid misinterpretation or miss proper detection. In some cases, especially when confronting transdiploic lesions, the use of functional imaging techniques such as MR DWI, MRS, and MR DSC-PWI can yield specific findings, which may help discriminate among otherwise undifferentiable lesions.

Imaging plays a growing pivotal role in the diagnostic process of skull vault lesions. The importance of this work lays on the need of radiologists to recognize and properly manage skull vault lesions. Beyond morphologic evaluation, this work adds the value of MR DWI, MRS, and MR DSC-PWI. Dominion of the subject may avoid undesirable diagnostic delays, expensive tests, and potentially harmful procedures in favor of prompt diagnostic, tests adequacy, and guided biopsies if needed.

## Data Availability

Data sharing is not applicable to this article as no datasets were generated or analyzed during the current study.

## Abbreviations

ADC: Apparent diffusion coefficient
CECT: Contrast-enhanced CT
CT: Computed tomography
DSC: Dynamic susceptibility contrast
DWI: Diffusion-weighted imaging
MRI: Magnetic resonance imaging
MRS: Magnetic resonance spectroscopy
NECT: Non-enhanced CT
PWI: Perfusion-weighted imaging
rCBV: Relative cerebral blood volume
ROI: Region of interest
SUVmax: Maximum standardized uptake value
TIC: Time-intensity curve
WI: Weighted images
